# Investigating the co-assembly of amphipathic peptides

**DOI:** 10.1039/d5fd00036j

**Published:** 2025-03-25

**Authors:** Zixuan Liu, Alberto Saiani, Aline F. Miller

**Affiliations:** a Department of Chemical Engineering & Manchester Institute of Biotechnology, School of Engineering, Faculty of Science and Engineering, The University of Manchester UK aline.miller@manchester.ac.uk +44 (0) 7714094668; b Division of Pharmacy and Optometry & Manchester Institute of Biotechnology, School of Health Sciences, Faculty of Biology, Medicine and Health, The University of Manchester UK

## Abstract

Self-assembling peptide hydrogels (SAPHs) are increasingly recognised for their potential in biomedical and bioelectronic applications, with recent work focusing on exploiting the understanding of molecular self-assembly across the length scales. The resulting soft hydrogel materials are typically formulated by exploiting the self-assembly of short peptides into fibrillar aggregates that entangle and associate into networks. As more complex systems are thought to be needed to accommodate the needs of various applications, the mixing of peptides to form mixed SAPHs has come to the fore as a potential approach to design new systems with tailored and functional properties. This strategy has raised the question of whether mixing peptides with different chemical structures results in co-assembly or the formation of distinct fibrillar aggregates. In this work, we have used the FITC/Dabcyl FRET pair to investigate the co-assembly of a set of amphipathic short peptides. Our results show that the occurrence of co-assembly is affected the peptides’ physicochemical properties, in particular solubility and hydrophobic residue side-group nature.

## Abstract 2

The co-assembly of amphipathic peptides is explored here and we show the physicochemical properties, the core hydrophobic residues’ side-group size and their packing, affect co-assembly occurrence into self-assembling peptide hydrogels.

## Graphical abstract

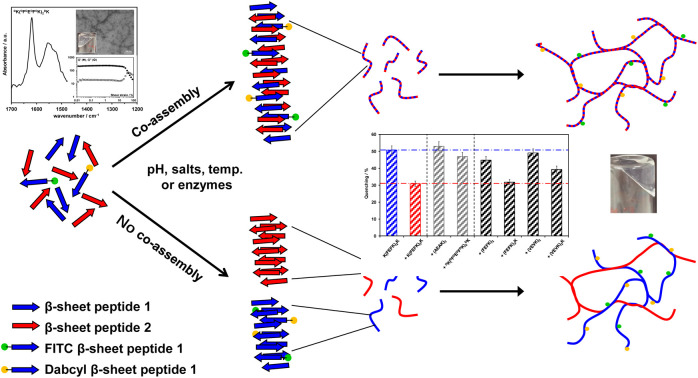

## Introduction

Self-assembling peptide hydrogels (SAPHs) have attracted significant interest over the last decade due to their potential for use in a broad range of biomedical applications, ranging from fully defined scaffolds for *in vitro* cell and organoid culture to *in vivo* delivery vehicles for molecular drugs, biologics and cells.^1,2^ These soft materials are formulated by exploiting the self-assembly properties of short peptides. A variety of peptide designs that self-assemble into fibres and form hydrogels have been developed over time.^3–6^ One particular design that has attracted significant interest is based on the alternation of hydrophobic and hydrophilic residues. Originally developed by Zhang and coworkers,^7,8^ these short peptides, typically 4 to 16 amino acids long, have the ability to self-assemble into β-sheet-rich fibres that, above a critical gelation concentration, associate and/or entangle to form hydrogels.^9–11^ This particular family of SAPHs has been shown to be biocompatible,^12–14^ non-immunogenic^12,15,16^ and allow the 3D culture of a broad variety of cells and organoids.^17–23^ Recently we have also shown how the physicochemical properties of these peptides, and therefore of the fibres forming the hydrogels, can have a strong impact on cell behaviour and fate.^24–26^

As more complex systems are thought to accommodate the needs of various applications, the integration of different peptides to form mixed SAPHs has come to the fore as a potential approach to design new systems with tailored properties.^27–29^ This strategy has raised the question of when mixing peptides with different chemical structures together, whether co-assembly occurs or distinct fibrillar aggregates form. The issue of short peptide co-assembly has been the subject of a number of recent works showing that peptide properties such as pH and temperature of self-assembly, molecular structure and formulation pathway can dictate whether co-assembly occurs.^30–34^

**Fig. 1:**
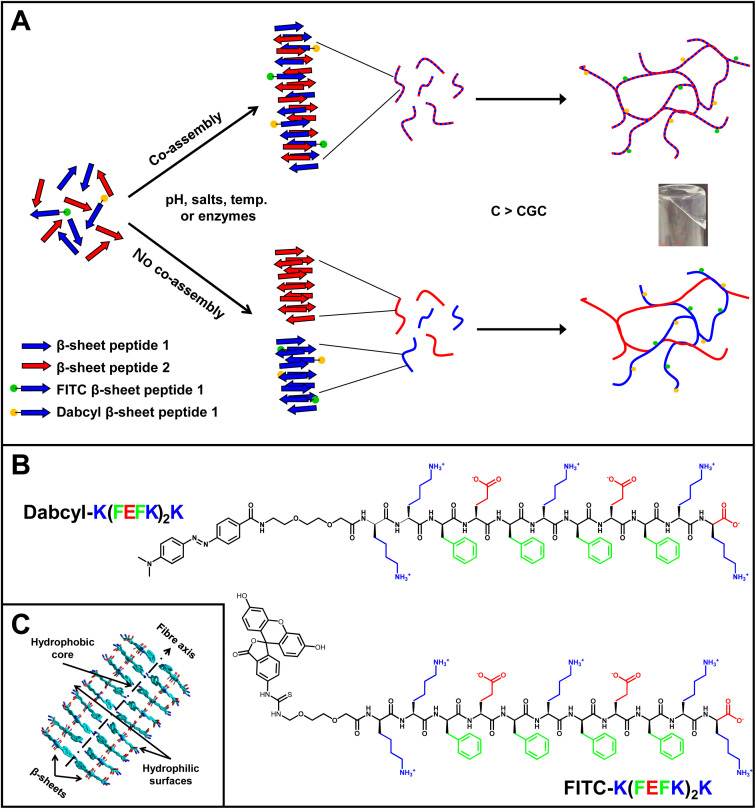
(A) Schematic representation of the self-assembly pathway of our peptides and labelling approach used to investigate co-assembly; if co-assembly occurs, the average spacing between FITC and Dabcyl chromophores increases resulting in an increase in fluorescence. (B) Chemical structure of labelled peptides used. (C) Schematic representation of the β-sheet fibres’ molecular packing.

In the present work, we explore whether the Förster resonance energy transfer (FRET) pair fluorescein isothiocyanate (FITC)/4-(dimethylaminoazo) benzene-4-carboxylic acid (Dabcyl) could be used to investigate the co-assembly of our family of amphipathic oligopeptides. Dabcyl is known to quench the fluorescence of FITC when the distance between the two chromophores is in the 1 to 10 nm range.^35^ In Fig. 1A, a schematic representation of the strategy used is shown. We chose K(FEFK)_2_K (F: phenylalanine; E: glutamic acid; K: lysine) as our base peptide (peptide 1 in Fig. 1A). This peptide is known to self-assemble into β-sheet-rich fibres that entangle and associate to form transparent and stable hydrogels at pH 7.^36^ This sequence has also been used in previous work to design hydrogels for a range of biomedical applications, including drug delivery^37^ and cell culture.^38^ We conjugated both FITC and Dabcyl to the end N-terminus of K(FEFK)_2_K *via* a short linker to create FITC–K–K(FEFK)_2_K and Dabcyl–K–K(FEFK)_2_K (Fig. 1B). The hypothesis was that when adding a second peptide (peptide 2 in Fig. 1A) during hydrogel formulation, if co-assembly occurs, the average spacing between FITC and Dabcyl chromophores along the peptide fibres increases resulting in an increase in the overall sample fluorescence. On the other hand, if no co-assembly occurs and distinct fibrillar aggregates are formed, the overall average spacing between FITC and Dabcyl chromophores will remain unchanged resulting in an unchanged overall sample fluorescence.

Mixed SAPHs were created by mixing K(FEFK)_2_K with six other peptides whose sequences were built using the same design but had difference physicochemical properties. (AEAK)_2_ (A: alanine) was shown in a previous study to be soluble due to its reduced hydrophobicity (replacement of F by A) and therefore did not self-assemble nor form hydrogels at pH 7.^39^^D^K(^D^F^D^E^D^F^D^K)_2_^D^K has the opposite chirality to the base peptide, which was synthesised using l-amino acids, and therefore is not expected to co-assemble with K(FEFK)_2_K but to form separate fibrillar aggregates.^33^ These two peptides were used as negative controls, as in both cases co-assembly was not expected to occur. As the positive control, the base peptide K(FEFK)_2_K was used as the secondary peptide, as in this case co-assembly will of course occur.

We then used the following four self-assembling peptides: (FEFK)_2_, (FEFK)_2_K, (VEVK)_2_ and (VEVK)_2_K (V: valine) to explore the effect that the physicochemical properties of the peptide sequence had on the propensity of co-assembly. These four peptides have been used by our group in previous studies and have all been shown to form on their own similar β-sheet-rich fibres and hydrogels.^36,39–41^ (FEFK)_2_ and (VEVK)_2_ will carry an overall neutral charge at pH 7 while (VEVK)_2_K and (FEFK)_2_K will carry an overall +1 charge. The replacement of F by V in the main sequence will result in a decrease in the overall hydrophobicity of the peptide and is also thought to affect the molecular packing in the core of the β-sheet fibre. A schematic representation of the self-assembled fibre structure formed by this family of peptides is shown in Fig. 1C. Due to the design used, all the hydrophobic side-chain residues are thought to be located within the core of the β-sheet fibre, which is formed by two cross-β-sheets coming together to bury their hydrophobic faces.^36,42,43^

## Materials and methods

### Materials

All peptides used in this study were purchased from LifeTein Inc. (USA) as HCl salts with a nominal sequence purity of 95%. Peptide sequence purities were confirmed by reverse-phase high-performance liquid chromatography and mass spectroscopy by the supplier. All other chemicals were purchased from Sigma-Aldrich and Merck and used as received.

### Self-assembling peptide hydrogels’ (SAPHs) formulation

SAPHs at pH 7 were prepared in 5 mL batches by dissolving the required amount of peptide and conjugated peptide powders in 3.5 mL of HPLC grade water using vigorous vortex mixing for 60 s. The samples initial pH ranged from 2.1 to 2.3. The pH was then adjusted to 7 by the stepwise addition of a 0.5 M NaOH solution. The samples were mixed vigorously and gently centrifuged, if necessary, to remove any bubbles after each NaOH addition. Once pH 7 was achieved, the required additional HPLC-grade water was added to achieve the target batch volume and concentration. The samples were then mixed a final time and stored (for at least 12 h) in a fridge before use. Diluted SAPH samples were prepared by adding the required volume, to achieve the targeted dilution, of HPLC-grade water on top of the pre-prepared SAPH and mixing using a vortex. The samples were then once again stored in a fridge for at least 12 hours before use.

### Fluorescence

Sample fluorescence emission was measured using an Agilent Cary Eclipse Fluorescence Spectrometer at 520 nm (excitation wavelength: 490 nm). 2 mL of each sample was placed in a cuvette with a 2 cm path length. The sample quenching percentage was defined as the ratio of the sample fluorescence with added Dabcyl–K–K(FEFK)_2_K to the sample’s fluorescence without added Dabcyl–K(FEFK)_2_K.

### Characterization of ^D^K(^D^F^D^E^D^F^D^K)_2_^D^K SAPH

Attenuated Total Reflectance Fourier-Transform Infrared (ATR-FTIR) spectroscopy and transmission electron microscopy (TEM) were used to characterise the SAPH formed by ^D^K(^D^F^D^E^D^F^D^K)_2_^D^K at 7.5 mg mL^−1^. ATR-FTIR measurements were performed on a Bruker VERTEX 80 FTIR spectrometer equipped with a single-bounce diamond ATR accessory. A small drop of hydrogel was placed on the surface of the diamond and pressed into position using a spatula to ensure good contact between the hydrogel and the diamond surface. The beam path was purged with dry CO_2_-scrubbed air. The spectra were an average of 256 scans collected using a 4 cm^−1^ resolution. An HPLC-grade water spectrum was used as a background and subtracted from each sample spectrum. For TEM preparation, samples were diluted 20-fold and vigorously mixed with a vortex to maximise the separation of the fibres. A carbon-coated copper grid (400 mesh grid Electron Microscopy Sciences, Hatfield, Pennsylvania, USA) was glow discharged and charged negatively. The carbon copper grid was then placed sequentially on a 10 μL sample droplet for 60 s, a 10 μl droplet of HPLC-grade water for 10 s, a 10 μL droplet of 5% uranyl acetate solution for 30 s, and finally, on a 10 μL droplet of HPLC-grade water for 10 s. After each step, excess liquid was drained off using lint-free tissue (90 mm Whatman 1). The grid was then left to air-dry for 2 to 5 min. TEM images were taken using an FEI Tecnai12 BioTwin transmission electron microscope running at 100 kV and equipped with a Gatan Orius SC1000A CCD camera. Rheological measurements were performed using a Discovery Hybrid 2 (DHR-2) rheometer from TA Instruments (New Castle, Delaware, USA) using a 20 mm parallel plate geometry and a 500 μm gap. The hydrogel (200 μL) was pipetted onto the rheometer's static bottom plate and the rheometer top plate lowered to the desired gap size. The sample was covered with a solvent trap to avoid evaporation. Strain sweep experiments were performed at 1 Hz in the 0.01–100% strain range.

## Results and discussion

First, we investigated the effect that adding FITC–K–K(FEFK)_2_K to a range of mixed SAPHs had on the samples' overall fluorescence. All mixed SAPHs were prepared with a base peptide concentration of 3.75 mg mL^−1^ and a total peptide (base + secondary peptide) concentration of 7.5 mg mL^−1^. This gave a base to secondary peptide mass ratio of 1 : 1.

**Fig. 2:**
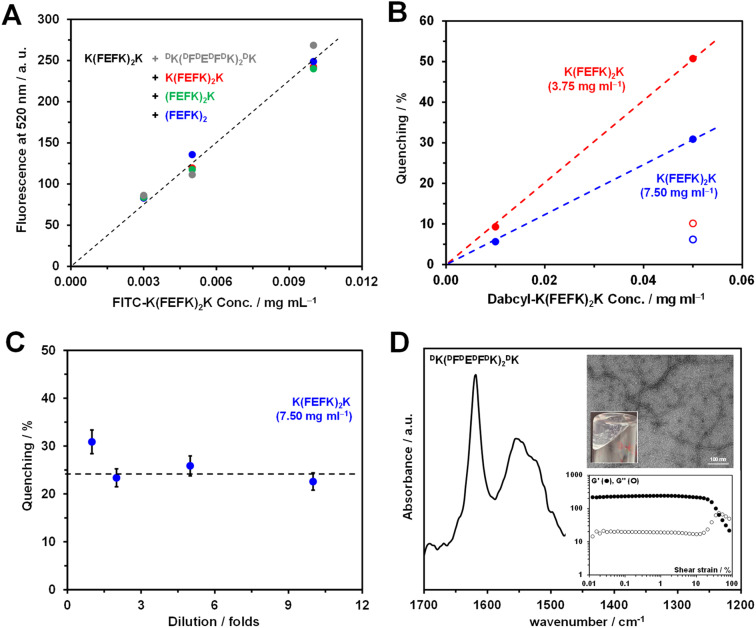
(A) FITC–K–K(FEFK)_2_K concentration *vs.* mixed SAPHs’ fluorescence; total sample peptide concentration: 7.5 mg mL^−1^; base peptide to secondary peptide mass ratio: 1 : 1. (B) Base SAPHs’ fluorescence quenching percentage [(sample fluorescence with Dabcyl–K–K(FEFK)_2_K / sample fluorescence without Dabcyl–K–K(FEFK)_2_K) × 100] *vs.* Dabcyl–K–K(FEFK)_2_K concentration; K(FEFK)_2_K base SAPHs’ peptide concentration: 3.75 mg mL^−1^ (red) and 7.5 mg mL^−1^ (blue); FITC–K–K(FEFK)_2_K concentration: 0.01 mg mL^−1^; open symbols: quenching percentages normalised to 0.01 mg mL^−1^ Dabcyl–K–K(FEFK)_2_K concentration. (C) Base SAPH quenching percentage *vs.* dilution fold; base SAPH peptide concentration: 7.5 mg mL^−1^. (D) FTIR spectrum of ^D^K(^D^F^D^E^D^F^D^K)_2_^D^K SAPH prepared at 7.5 mg mL^−1^; top inset: TEM image of fibres formed and photograph of hydrogel; bottom inset: storage (*G*′) and loss (*G*′′) moduli *vs.* shear strain.

First, we added FITC–K–K(FEFK)_2_K only to the mixed SAPHs. As can be seen from Fig. 2A, the samples' fluorescence increased linearly with increasing FITC–K–K(FEFK)_2_K content, and this was independent of which second peptide was used to create the mixed SAPHs. This result clearly shows that the secondary peptide does not affect the sample fluorescence in any way, and therefore the fluorescence of the mixed SAPHs can be directly compared.

We then chose the sample containing the highest concentration (0.01 mg mL^−1^) of the FITC–K–K(FEFK)_2_K probe and investigated the effect that adding Dabcyl–K–K(FEFK)_2_K to the base SAPHs had on the samples' fluorescence *via* the quenching percentage, which was defined as:1
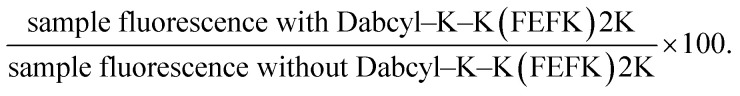


Two K(FEFK)_2_K concentrations were used to make these SAPHs: 3.75 mg mL^−1^ for the initial concentration of the ‘base’ formulation, and 7.5 mg mL^−1^, which is the total peptide concentration of the ‘mixed’ formulations. As can be seen from Fig. 2B, the quenching percentage was directly proportional to the Dabcyl–K–K(FEFK)_2_K concentration in the range investigated. As expected, the quenching percentages for the 3.75 mg mL^−1^ base SAPHs were found to be significantly higher compared to the 7.5 mg mL^−1^ base SAPHs, which reflects the increase in distance between the fluorophore (FITC) and quencher (Dabcyl) moieties at the higher peptide concentration. In addition, the overall quenching percentage results for both SAPHs were found to be directly proportional to the Dabcyl–K–K(FEFK)_2_K concentration (Fig. 2B open symbols: concentration normalised values). These results confirm that both labelled peptides co-assembled with the base peptide and that the approach used allows the investigation of co-assembly in these systems. The quenching percentages of these two base SAPH formulations represent the minimum and the maximum quenching expected when co-assembly does, and does not, occur, respectively.

It should be noted that FITC quenching can have an intra-fibre (Dabcyl situated along the same peptide fibre) or an inter-fibre (Dabcyl located on a different fibre) origin. In order for this methodology to be reliable, the quenching observed should mainly be due to intra-fibre quenching events. We have shown in previous works that the mesh size in these networks is in the 10–30 nm range depending on peptide concentration^42,43^ and therefore inter-fibre quenching events are a possibility. To investigate this point further, we diluted the 7.5 mg mL^−1^ base SAPH formulation 2-, 5- and 10-fold and measured the resulting samples' fluorescence. By diluting the full sample, the network mesh size/distance between peptide fibres is increased^42,43^ resulting in an increase in distance between the FITC and Dabcyl chromophores located on different fibres. As a result, inter-fibre quenching events are expected to decrease with increasing dilution. On the other hand, along the peptide fibres, the ratios between the FITC- and Dabcyl-conjugated peptides and the base peptide remain the same, and therefore the distance between FITC and Dabcyl along the fibre remains unchanged. As a result, intra-fibre quenching events are not expected to be affected by dilution. Consequently, in the absence of inter-fibre quenching events, the diluted samples’ quenching percentages are expected to remain unchanged. As can been seen from Fig. 2C, when diluting 2-fold the quenching percentage was found to decrease slightly by ∼8% and then remained unchanged when further dilutions were performed. This result shows that at 7.5 mg mL^−1^ concentration some inter-fibre quenching events indeed occur, but overall the quenching observed is mainly due to intra-fibre quenching events.

Next, we investigated how the sample's fluorescence changed when adding the two negative control peptides – (AEAK)_2_ and ^D^K(^D^F^D^E^D^F^D^K)_2_^D^K – to the base peptide. As mentioned above, neither of these two peptides are expected to co-assemble with the base peptide. For the latter peptide, ^D^K(^D^F^D^E^D^F^D^K)_2_^D^K, we first confirmed that it indeed self-assembled into β-sheet-rich fibres and formed hydrogels on its own. In Fig. 2D, the FTIR spectrum obtained for the hydrogel prepared using this peptide at 7.5 mg mL^−1^ is shown. A strong peak at ∼1630 cm^−1^ and a weaker peak at ∼1696 cm^−1^ typical of the adoption of cross-β-sheet conformations by this family of peptides are observed.^44,45^ The TEM image (Fig. 2D, top inset) clearly shows the formation of thin semi-flexible fibres with similar dimensions, ∼3 to 4 nm in diameter, to those observed for the base peptide.^36^ Formation of a hydrogel was confirmed visually (photograph in Fig. 2D, top inset) and mechanically through shear rheometry (Fig. 2D, bottom inset). As is typical for solid-like materials, the SAPH storage modulus (*G*′) was found to be one order of magnitude larger than then the loss modulus (*G*′′) and both were found to be constant up to 15% shear strain.

**Fig. 3:**
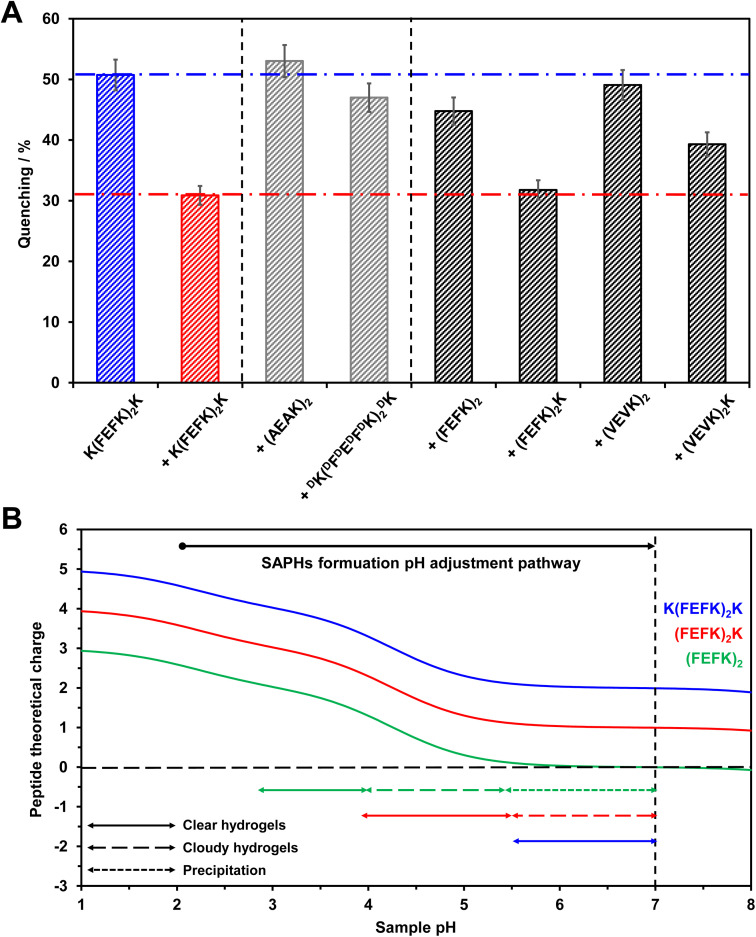
(A) Fluorescence quenching percentage *vs.* peptide used to prepare mixed SAPHs. (B) Theoretical overall peptide charge and sample phase behaviour *vs.* formulation pH pathway. The overall charge carried by a peptide was calculated using the following equation: 
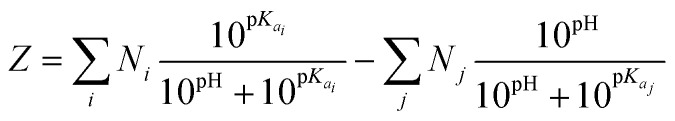
, where *N*_*i*/*j*_ are the numbers, and p*K*_a_*i*/*j*__ are the p*K*_a_ values, of the basic (*i* – p*K*_a_ > 7) and acidic (*j* – p*K*_a_ < 7) groups present, respectively. The ionic groups present on the peptides are carboxylic acid (COOH/COO^−^) at the C-terminus (theoretical p*K*_a_ of 2.18) and on the glutamic acid side chains (theoretical p*K*_a_ of 4.25), and amine (NH_3_^+^/NH_2_) at the N-terminus (theoretical p*K*_a_ values of 8.95 and 9.13 on the K and F sides, respectively) and on the lysine side chains (theoretical p*K*_a_ of 10.53).

As can be seen from Fig. 3A, when adding (AEAK)_2_ to the base peptide, no change in quenching percentage compared to the base SAPH formulation at 3.75 mg mL^−1^ was observed, confirming that, as expected, no co-assembly occurred. As mentioned above, (AEAK)_2_ is soluble and does not self-assemble^42^ and is therefore simply molecularly dissolved in the water phase of the hydrogel. For the ^D^K(^D^F^D^E^D^F^D^K)_2_^D^K peptide, a slight decrease in quenching percentage (∼4%, Fig. 3A) was observed compared to the base SAPH formulation at 7.5 mg mL^−1^. In this case too, no co-assembly was expected, but this peptide, as shown above, does self-assemble into β-sheet-rich fibres. As a result, two independent interpenetrating fibrillar networks are thought to form. The slight decrease observed in quenching percentage is thought to be due to the presence of the ^D^K(^D^F^D^E^D^F^D^K)_2_^D^K fibrillar network limiting inter-fibre quenching events.

We then went on to investigate the change in the samples' fluorescence when the peptides (FEFK)_2_ and (FEFK)_2_K were used to create mixed SAPHs. It should be noted that, when formulating the SAPHs, peptide powders were mixed first before water was added, resulting in the formation of acidic peptide solutions (pH ∼2.2). Self-assembly and hydrogelation were then triggered by adjusting the pH of the solutions through stepwise addition of NaOH and vortexing after each step to ensure homogeneity (see the Materials and methods section for more details). These three peptides (base peptide + the two secondary peptides) were designed with the same sequence core – (FEFK)_2_ – but with different numbers of K tagged at the peptide ends – none, one and two – leading to varying peptide charge *vs.* pH profiles (Fig. 3B). As we have shown in our previous work, transparent hydrogel formation in these systems tends to occur when the overall charge carried by the peptide is in the ∼+2.5 to +1.5 range.^36,46^ As can be seen from Fig. 3B, this leads to different phase behaviour *vs.* pH profiles for each peptide. The base peptide, K(FEFK)_2_K, formed transparent SAPHs for pH > ∼5.5. For (FEFK)_2_K, transparent SAPHs were found to form in the pH range of ∼4 to 5.5. Above ∼5.5, the charge carried by the peptide is approaching +1 resulting in the formation of cloudy SAPHs. As discussed in our previous work, this is due to the formation of large fibre aggregates that scatter light. Indeed, as electrostatic repulsion between fibres decreases with increasing pH, hydrophobic attractive inter-fibre interactions become dominant leading to fibre aggregation. This effect is even more marked for (FEFK)_2_. In this case, transparent SAPHs formed in the pH range of ∼3 to 4, while cloudy SAPHs form in the pH range of ∼4 to 5.5. Above 5.5, the overall charge carried by the peptide becomes neutral and hydrophobic interactions dominate leading to fibre aggregation and precipitation.^36,46^

As can be seen from Fig. 3A, the overall phase behaviour of the peptide affects the occurrence of co-assembly. For (FEFK)_2_K, there is no change in the sample's fluorescence quenching percentage compared to the base SAPHs at 7.5 mg mL^−1^. This indicates that co-assembly did occur in this case. It is thought that the decrease in peptide hydrophobicity at pH 7 resulting from the presence of the additional K allows the gelation ranges of this and the base peptide to overlap, enabling co-assembly to occur in the mixed SAPH formulation. For (FEFK)_2_, the quenching percentage was similar to the value observed for the ^D^K(^D^F^D^E^D^F^D^K)_2_^D^K mixed SAPH, suggesting that in this case no, or very limited, co-assembly occurred. It is thought that, for this latter peptide, co-assembly is prevented as aggregation and precipitation occurs in the pH range where the base peptide on its own is known to self-assemble and form a hydrogel.

Finally, we investigated whether co-assembly occurred when (VEVK)_2_ and (VEVK)_2_K were used to formulate the mixed SAPHs. A similar phase behaviour *vs.* pH was observed for these two systems as for their F-equivalent peptides discussed above. No change in quenching percentage was observed for the (VEVK)_2_ mixed SAPH compared to the ^D^K(^D^F^D^E^D^F^D^K)_2_^D^K mixed SAPH, once again suggesting that no co-assembly occurred. Here too, the poor solubility of this peptide and the fibres formed at pH 7 are thought to prevent any co-assembly with the base peptide. Interestingly though, (VEVK)_2_K was found to have an intermediate behaviour, with the quenching percentage of this mixed SAPH (∼40%) being in between the quenching percentages expected when co-assembly did (∼30%) and did not (∼50%) occur. This result suggests that partial co-assembly may occur. As for (FEFK)_2_K, no solubility issues were expected to prevent co-assembly; therefore, it is thought that the replacement of F by V is affecting co-assembly. The side groups of F and V have different volumes and sizes and therefore are expected to lead to different packing constrains in the core of the beta-sheet fibres. This is thought to lead to partial co-assembly and formation of ‘blocky’ assemblies, where blocks of base peptide and (VEVK)_2_K alternate along the same fibre. This result is in agreement with our previous work where we showed that changing one F-residue side group to 2,3-dibromomaleimide in (FEFK)_2_K can lead to a change in fibre morphology. This was thought to be due to a change in packing constraints in the fibre core resulting from introduction of the modification.^39^

## Conclusions

Here, we have investigated the possibility of using the FRET pair FITC/Dabcyl to investigate the co-assembly of short amphipathic oligopeptides into mixed SAPH formulations. Our results clearly show that this methodology can be used to explore the co-assembly phenomenon, and that the occurrence of co-assembly is affected by the peptides’ physicochemical properties, in particular the overall charge carried by the peptide and the resulting phase behaviour. Interestingly, our work also shows that the nature of the hydrophobic residues’ side group and therefore the packing on the fibre core also affects co-assembly. Indeed, when using (VEVK)_2_K peptides to prepare a mixed SAPH with our K(FEFK)_2_K base peptide, the results suggest formation of ‘blocky’ assemblies, with alternation of different peptide self-assembled blocks along the same fibre.

This work clearly shows how peptide design plays a key role in defining co-assembly occurrence in these systems and how mixed SAPHs can be a promising route to build novel hydrogel systems. Developing our molecular understanding of co-assembly in these systems is key to designing hydrogels with novel properties and functionalities.

## Conflicts of interest

There are no conflicts to declare.

## Data Availability

All research data supporting this publication are directly available within this publication.
